# The complete chloroplast genome of *Sloanea hemsleyana*

**DOI:** 10.1080/23802359.2022.2090290

**Published:** 2022-07-04

**Authors:** Xia Yang, Yu-Xue Zhao, Jia-Min Zhu, Liu-Yan Wu, Zhi-Ping Chen

**Affiliations:** a Northwest A&F University Forestry college, Xianyang, China; b Guizhou Academic of Forestry, Guiyang, China; c Guizhou Institute of Walnut, Guiyang, China

**Keywords:** *Sloanea hemsleyana*, chloroplast genome, phylogenetic analysis

## Abstract

*Sloanea hemsleyana* is a potential commercial and oil tree species. This study is the first to report and analyze complete chloroplast genome sequences of *S. hemsleyana* as a genomic resource for conservation purposes. The chloroplast genome is 158,085 bp in length and consisted of a large single-copy (LSC) region (88,446 bp), and a small single-copy (SSC) region (17,659 bp), separated by a pair of inverted repeats (IR) regions (25,990 bp). It contains 108 genes, with 74 protein-coding genes, 30 tRNA genes, and 4 rRNA genes. Phylogenetic analysis revealed that *S. hemsleyana* was most closely related to *S. sinensis.*

*Sloanea hemsleyana* (Ito) Rehd. et Wils.,1916, a member of the Elaeocarpaceae family, is a species of evergreen tree widely distributed in Hubei, Hunan, Yunnan, Sichuan, Guizhou and Guangxi Provinces in China. It is an excellent commercial tree species that provided wood suitable for construction, and its oil-rich seeds can be used to make biodiesel (Jia and Mi 1978). To date, only the complete chloroplast sequence of *S. sinensis* has been reported (Weng et al. 2021). Therefore, sequencing of the complete *S. hemsleyana* chloroplast genome may lead to an exhaustive understanding of photosynthesis and resulting wood production in them.

To obtain total *S. hemsleyana* chloroplast DNA, fresh leaf samples were collected from Guizhou Academy of Forestry (106°14′14″E,26°29′57″N), in Guizhou Province, China, according to ‘the methods collecting for plant samples of Guizhou Academy of Forestry’ (http://www.gzslky.com/). A specimen was deposited at the Guizhou Academy of Forestry (http://www.gzslky.com/, Zhi-Ping Chen and chzhping 723@163.com) under the voucher number 09017362. The total genomic DNA was extracted from fresh leaves using a modified CTAB method (Li et al. 2013) and paired end libraries were prepared using the NEBNext Ultra DNA Library Prep Kit., with 150 bp randomly interrupted using the Covaris ultrasonic breaker for library construction. The constructed library was sequenced using Illumina NovaSeq 6000, and approximately 1.2 GB of raw reads were generated. The resultant data were filtered using script in the cluster (default parameters: -L 5, -*p* 0.5, -N 0.1). The chloroplast genome was assembled using MITObim v1.9(Hahn et al. 2013), the genes were annotated using the online tool OGDRAW (https://chlorobox.mpimp-golm.mpg.de/OGDraw.html) (Lohse et al. 2013), and the annotated genome was deposited in the NCBI GenBank under the accession number MZ664554. All experiments in this study were permitted by the Forestry Bureau of Guizhou Province.

The complete chloroplast genome sequence of *S. hemsleyana* (GenBank accession MZ664554) was 158,085 bp in length, with LSC region of 88,446 bp, SSC region of 17,659 bp, and a pair of inverted repeats of 25,990 bp. The total GC content was ∼37.2%, whereas the GC content of the LSC, SSC and IRs regions were ∼36.0%, ∼31.4% and ∼42.9%, respectively. A total of 108 gene species were annotated, including 74 protein-coding genes, 30 tRNA genes, and 4 rRNA genes. Among these genes, 18 were duplicated in the IR regions,16 harbored a single intron, and 2(*clpP* and *ycf3*) contained double introns.

To reveal the phylogenetic position of *S. hemsleyana*, 16 complete chloroplast genomes were obtained from GenBank, and the sister group Malpighiales was used as an out group. All sequences were aligned using TOPALi v2.5 (Milne et al. 2009), and phylogenetic analysis was conducted using the maximum-likelihood method using MrBayes v3.1.2 (Ronquist and Huelsenbeck 2003). The phylogenetic analysis showed that *S. hemsleyana* was closely related to *S. sinensis* (Figure 1). The complete *S. hemsleyana* chloroplast genome can be future used for genetic engineering, population, and phylogentic studies of the Elaeocarpaceae family.

**Figure 1.**
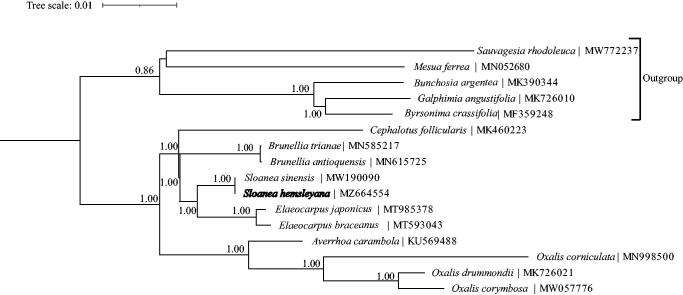
Maximum-likelihood tree based on 11 complete chloroplast genomes in Oxalidales. *Sauvagesia rhodoleuca, Mesua ferrea, Bunchosia argentea, Byrsonima crassifolia* and *Galphimia angustifolia* as out group, bootstrap support value near the branch.

## Author contributions

Xia Yang and Yu-Xue Zhao are the experimental designers and executors of the experimental research, completing the data analysis and writing the first draft of the paper; Jia-Min Zhu and Liu-Yan Wu participated in the experimental design and analysis of experimental results; Zhi-ping Chen is the creator and person in charge of the project, directing experimental design, data analysis, essay writing and revision. All authors have read and agreed to the final text.

## Data Availability

The genome sequence data that support the findings of this study are openly available in GenBank of NCBI at [https://www.ncbi.nlm.nih.gov] under the accession no.MZ664554. The associated BioProject, SRA, and Bio-Sample numbers are PRJNA769407, SRR16234991, and SAMN22132357, respectively.
